# Y_0.76_Ho_0.24_FeGe_2_O_7_: a new member of thortveitite-like layered compounds

**DOI:** 10.1107/S1600536809020467

**Published:** 2009-06-06

**Authors:** Ivonne Rosales, Elizabeth Chavira, Eligio Orozco, Lauro Bucio

**Affiliations:** a Instituto de Física, Universidad Nacional Autónoma de México, AP 20-364, 01000 México D.F., Mexico; b Instituto de Investigaciones en Materiales, Universidad Nacional Autónoma de México, AP 70-360, 01000 México D.F., Mexico

## Abstract

Y_0.76_Ho_0.24_FeGe_2_O_7_ (yttrium holmium iron digermanate) was synthesized by solid-state reaction at 1573 K. This thortveitite-like compound presents a crystallographic group–subgroup isotranslational (*klassengleiche*) relation with some other pyrogermanates, such as FeInGe_2_O_7_, In_1.08_Gd_0.92_Ge_2_O_7_ and InYGe_2_O_7_, which are configurationally isotypic with the Sc_2_Si_2_O_7_ thortveitite structure first reported by Zachariasen [(1930 ▶). *Z. Kristallogr.* **73**, 1–6]. Holmium cations share with yttrium the 4*f* Wyckoff position at the center of a seven-coordinated pentagonal bipyramid, while Fe atoms also occupy one site with Wyckoff position 4*f* at the center of the octahedron. All these sites have the point symmetry *C*_1_. Two types of Ge_2_O_7_ diorthogroups with point symmetry *C*_1*h*_ are present in the structure, each one of them defining a layer type which alternates with the other. These diorthogroups have their tetrahedral groups in an eclipsed conformation.

## Related literature

The method of preparation was based on work published by Cascales *et al.* (1998*b* ▶). For related structures, see: Zachariasen (1930 ▶); Cascales *et al.* (1998*a* ▶,*b* ▶, 2002 ▶); Bucio *et al.* (2001 ▶); Redhammer *et al.* (2007 ▶).

## Experimental

### 

#### Crystal data

Y_0.76_Ho_0.24_FeGe_2_O_7_
*M* *_r_* = 420.17
Monoclinic, 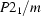

*a* = 9.6496 (2) Å
*b* = 8.5073 (2) Å
*c* = 6.6712 (2) Å
β = 100.621 (1)°
*V* = 538.27 (2) Å^3^
*Z* = 4
Cu *K*α radiation
*T* = 295 K
Specimen shape: flat sheet
20 × 20 × 0.2 mm
Specimen prepared at 1573 K
Particle morphology: spherical, brown

#### Data collection

Bruker Advance D8 diffractometer
Specimen mounting: packed powder sample container
Specimen mounted in reflection mode
Scan method: step
2θ_min_ = 10, 2θ_max_ = 80.0°
Increment in 2θ = 0.02°

#### Refinement

*R*_p_ = 0.07
*R*_wp_ = 0.09
*R*_exp_ = 0.06
*R*_B_ = 0.03
*S* = 1.53
Wavelength of incident radiation: 1.54175 Å
Profile function: pseudo-Voigt modified by Thompson *et al.* (1987 ▶)
843 reflections
60 parameters

### 

Data collection: *DIFFRAC/AT* (Siemens, 1993 ▶); cell refinement: *DICVOL91* (Boultif & Louër, 1991 ▶); data reduction: *FULLPROF* (Rodríguez-Carvajal, 2006 ▶); program(s) used to solve structure: coordinates taken from an isotypic compound (Cascales *et al.*, 2002 ▶) show [/query]>; program(s) used to refine structure: *FULLPROF* ; molecular graphics: *ATOMS* (Dowty, 2000 ▶); software used to prepare material for publication: *ATOMS*.

## Supplementary Material

Crystal structure: contains datablocks global, I. DOI: 10.1107/S1600536809020467/br2109sup1.cif

Rietveld powder data: contains datablocks I. DOI: 10.1107/S1600536809020467/br2109Isup2.rtv

Additional supplementary materials:  crystallographic information; 3D view; checkCIF report

## Figures and Tables

**Table:** 

Ge1—O4^i^	1.70 (3)
Ge1—O9^ii^	1.82 (3)
Ge1—O10	1.78 (3)
Ge1—O10^iii^	1.78 (3)
Ge2—O1	1.69 (3)
Ge2—O1^iv^	1.69 (3)
Ge2—O4	1.98 (3)
Ge2—O8	1.78 (4)
Ge3—O3^v^	1.77 (4)
Ge3—O5	1.82 (3)
Ge3—O5^iv^	1.82 (3)
Ge3—O6^vi^	1.76 (6)
Ge4—O2^vii^	1.67 (3)
Ge4—O3^vii^	1.73 (4)
Ge4—O7	1.87 (3)
Ge4—O7^iv^	1.87 (3)

**Table:** 

Ge1^viii^—O4—Ge2	127.7 (16)
Ge3^ix^—O3—Ge4^vi^	165 (2)

Symmetry codes: (i) 
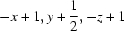
; (ii) 
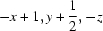
; (iii) 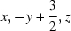; (iv) 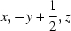; (v) 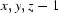; (vi) 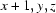; (vii) 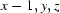; (viii) 
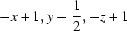
; (ix) 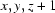.

**Fig. 1.**
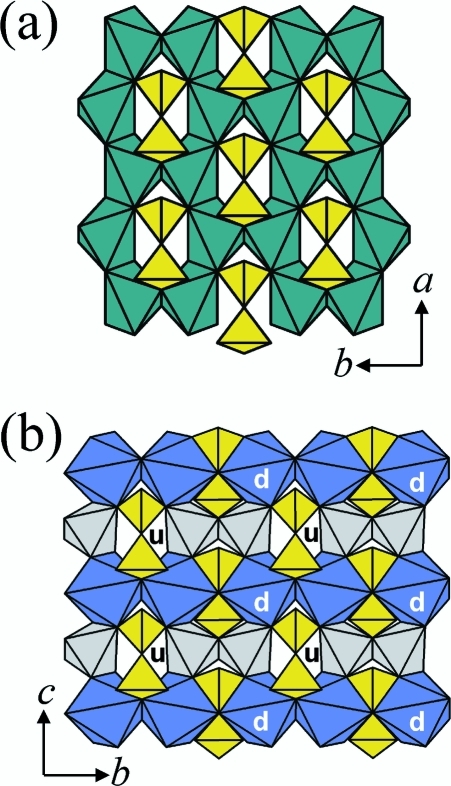
(a) View of a layer in the thortveitite structure (ab projection). Diorthogroups X2O7 are represented as yellow tetrahedra and MO6 octahedra in dark cyan. (b) View of thortveitite-like structure of Y0.76Ho0.24FeGe2O7 (ac projection). Diorthogroups Ge2O7 are represented as yellow tetrahedra, while FeO6 octahedra appear in light gray, and (Y,Ho)O7 polyhedra (medium slate blue). The bridging O atoms of Ge2O7 groups are displaced up (u) and down (d) normal to the cb plane from its original position in thortveitite.

**Fig. 2.**
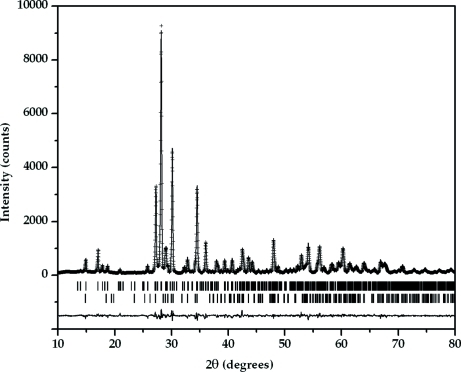
Rietveld refinement for Y0.76Ho0.24FeGe2O7 X-ray diffraction data. Observed (crosses), calculated (solid line) and difference (bottom trace) plots are represented; vertical marks correspond to the allowed Bragg reflections for Y0.76Ho0.24FeGe2O7 (top) and Y2Ge2O7 (bottom).

## supplementary crystallographic information

### Comment

The crystal structure of the original thortveitite was first reported by Zachariasen (1930) and has the formula Sc_2_Si_2_O_7_. In this silicate, the substitution of silicon has given rise to germanates, phosphates, arsenates and vanadates which present layered structures.

The ionic substitution from Si to Ge and Sc to trivalent metals and rare earths in thortveitite, give often germanates with thortveitite-like crystal structure RMGe_2_O_7_, where *R* and *M* represent cations of rare earths, transition metals, divalent or trivalent elements in octahedral coordination. The frameworks of these phases are built up from corner-sharing octahedra along *ab* planes forming a hexagonal disposition on layers interspersed with layers of Ge_2_O_7_ groups in staggered conformation (in  Fig. 1a the octahedra appear in dark cyan, while the Ge_2_O_7_ group in yellow color).

Some ionic substitutions give rise to seven-coordinated cations occupying the half of octahedral sites in the thortveitite structure. In such case, the generalized formula can be written as MR*X*_2_O_7_ where *X*_2_O_7_ is the same diorthogroup mentioned before presenting almost the same features as in thortveitite. The octahedral sites split in two new sites: half for cation *M* and other half for cation *R*, such as the cases for *R* = Y, Tb–Yb (Cascales *et al.*, 1998*a*,*b*, 2002). *M* remains with octahedral coordination while *R* changes its coordination to seven. In the present work we present the crystal structure of the new compound Y_1-_*_x_*Ho*_x_*FeGe_2_O_7_ with *x* = 0.24.

Fig. 1b show the crystal structure of Y_0.76_Ho_0.24_FeGe_2_O_7_ in which the bridging O atoms at the middle of the Ge_2_O_7_ diorthogroup are displaced up (*u*) or down (*d*) along a direction normal to the *cb* plane. RO_7_ polyhedra are connected alternately by either a vertex or an edge into chains along the *b* axis, Fig. 1b (medium slate blue colored polyhedra). In the same direction only isolated pairs of associated MO_6_ octahedra exist, as can be seen in the same figure (light gray octahedra). Flattened chains of RO_7_ polyhedra (in yellow) are linked in the c direction through pairs of MO_6_ octahedra with which they share edges forming layers running parallel to the *bc* crystal plane.

The most important feature in the structure previously described is the presence of Ge—O—Ge angles in the Ge_2_O_7_ group different from 180° giving rise to seven-coordinated cations in a half of the octahedral sites in the idealized thortveitite structure. The results of the Rietveld refinement established the presence of two crystalline phases for the method of synthesis used. The quantitative analysis gave 91.2 (8)% for Y_0.76_Ho_0.24_FeGe_2_O_7_ and 8.8 (3)% for Y_2_Ge_2_O_7_. With these results, the chemical reaction compatible with the quantitative analysis is:

4Y_2_O_3_ + Ho_2_O_3_ + 5Fe_2_O_3_ + 30GeO_2_→ 8.43Y_0.76_Ho_0.24_FeGe_2_O_7_ + 0.79Y_2_Ge_2_O_7_ + 11.56 GeO_2_ + 0.78Fe_2_O_3_.

### Experimental

The reactive mixture was prepared from Y_2_O_3_ (Aldrich.99.99%), Ho_2_O_3_ (Aldrich.99.9%), Fe_2_O_3_ (Aldrich.99.99%) and GeO_2_ (CERAC 99.999%) according to the method reported by Cascales *et al.* (1998*b*). This mixture was first powdered using an agate mortar; and then was heated in air in a tube furnace at 1573 K for 5 d with intermediate regrinding. At the end of the reaction, some vitreous phase impregnated and segregated at the bottom of the crucible was attributed to the presence of amorphous GeO_2_. Small amount of Fe_2_O_3_ was also detected as trace phase. The characterization of the bulk material by conventional X-ray powder diffraction data indicated two phases well crystallized. One of them showed reflections that were explained matching the isostructural phase YFeGe_2_O_7_ (PDF 01-072-6099) and the other one was identified as Y_2_Ge_2_O_7_ (PDF 38-288).

### Refinement

The structural model for YFeGe_2_O_7_ (ICSD 95935) was taken for start the Rietveld refinement of Y_1-_*_x_*Ho*_x_*FeGe_2_O_7_ with *x* = 1/5, while for the secondary phase, the data used for Y_2_Ge_2_O_7_ (ICSD 240989) was those reported by Redhammer *et al.* (2007). The Rietveld refinement was made using the Fullprof program (Rodríguez-Carvajal, 2006). A pseudo-Voigt function modified by Thompson *et al.* (1987) was chosen to generate the peak shape of the diffraction reflections. The following parameters were refined: zero point and scale factors, cell parameters, half-width profile parameters, overall temperature factors, preferred orientation, atomic coordinates, and asymmetries. For the Y_2_Ge_2_O_7 _phase no preferred orientation was considered, and the atomic coordinates were fixed to their starting values and an overall temperature factor was considered. The background was refined first by mean of a linear interpolation between 55 background points with adjustable heights. At the end of the refinement, the values for all of these heights of the background were fixed. The final Rietveld refinement of conventional diffraction pattern is shown in Figure 21.

### Crystal data

**Table:**

Y_0.76_Ho_0.24_FeGe_2_O_7_	*Z* = 4
*M**_r_* = 420.17	*F*(000) = 768.0
Monoclinic, *P*2_1_/*m*	*D*_x_ = 5.186 Mg m^−^^3^
Hall symbol: -P 2yb	Cu *K*α radiation, λ = 1.54175 Å
*a* = 9.6496 (2) Å	*T* = 295 K
*b* = 8.5073 (2) Å	brown
*c* = 6.6712 (2) Å	flat sheet, 20 × 20 mm
β = 100.621 (1)°	Specimen preparation: Prepared at 1573 K
*V* = 538.27 (2) Å^3^	

### Data collection

**Table:**

Bruker Advance D8 diffractometer	Data collection mode: reflection
Radiation source: sealed X-ray tube, Cu Kα	Scan method: step
graphite	2θ_min_ = 10°, 2θ_max_ = 80.00°, 2θ_step_ = 0.02°
Specimen mounting: packed powder sample container	

### Refinement

**Table:**

Least-squares matrix: full with fixed elements per cycle	3501 data points
*R*_p_ = 0.07	Profile function: pseudo-Voigt modified by Thompson *et al.* (1987)
*R*_wp_ = 0.09	60 parameters
*R*_exp_ = 0.06	Weighting scheme based on measured s.u.'s
*R*_Bragg_ = 0.03	(Δ/σ)_max_ = 0.02
*R*(*F*^2^) = 0.03	Background function: linear interpolation between a set background points with refinable heights
χ^2^ = 2.341	

### Fractional atomic coordinates and isotropic or equivalent isotropic displacement parameters (Å^2^)

**Table:**

	*x*	*y*	*z*	*U*_iso_*/*U*_eq_	Occ. (<1)
Y	0.7521 (11)	0.5406 (3)	0.7509 (10)	0.0120 (5)	0.76000
Ho	0.7521 (11)	0.5406 (3)	0.7509 (10)	0.0120 (5)	0.24000
Fe	0.7467 (15)	0.4473 (5)	0.2648 (16)	0.0120 (5)	
Ge1	0.5263 (14)	0.75000	0.0459 (17)	0.0120 (5)	
Ge2	0.5471 (13)	0.25000	0.4897 (14)	0.0120 (5)	
Ge3	0.9469 (13)	0.25000	0.0252 (15)	0.0120 (5)	
Ge4	0.0310 (13)	0.25000	0.5427 (18)	0.0120 (5)	
O1	0.627 (3)	0.427 (3)	0.499 (5)	0.0120 (5)	
O2	0.874 (4)	0.25000	0.389 (4)	0.0120 (5)	
O3	0.966 (3)	0.25000	0.766 (5)	0.0120 (5)	
O4	0.590 (3)	0.25000	0.792 (4)	0.0120 (5)	
O5	0.845 (3)	0.070 (3)	0.028 (4)	0.0120 (5)	
O6	0.130 (6)	0.25000	0.119 (6)	0.0120 (5)	
O7	0.150 (3)	0.083 (3)	0.502 (3)	0.0120 (5)	
O8	0.375 (5)	0.25000	0.334 (5)	0.0120 (5)	
O9	0.606 (4)	0.25000	0.186 (5)	0.0120 (5)	
O10	0.640 (3)	0.584 (3)	0.070 (3)	0.0120 (5)	

### Atomic displacement parameters (Å^2^)

**Table:**

	*U*^11^	*U*^22^	*U*^33^	*U*^12^	*U*^13^	*U*^23^
?	?	?	?	?	?	?

### Geometric parameters (Å, °)

**Table:**

Fe—O1	2.11 (4)	Ge1—O10	1.78 (3)
Fe—O2	2.15 (2)	Ge1—O10^vi^	1.78 (3)
Fe—O5^i^	1.99 (3)	Ge2—O1	1.69 (3)
Fe—O7^ii^	2.04 (2)	Ge2—O1^i^	1.69 (3)
Fe—O9	2.16 (2)	Ge2—O4	1.98 (3)
Fe—O10	1.90 (2)	Ge2—O8	1.78 (4)
Ho—O1	2.11 (3)	Ge3—O3^vii^	1.77 (4)
Ho—O4	2.96 (2)	Ge3—O5	1.82 (3)
Ho—O5^iii^	2.12 (3)	Ge3—O5^i^	1.82 (3)
Ho—O6^ii^	2.20 (3)	Ge3—O6^viii^	1.76 (6)
Ho—O7^ii^	2.11 (3)	Ge4—O2^ix^	1.67 (3)
Ho—O8^ii^	2.18 (3)	Ge4—O3^ix^	1.73 (4)
Ho—O10^iv^	2.59 (3)	Ge4—O7	1.87 (3)
Ge1—O4^ii^	1.70 (3)	Ge4—O7^i^	1.87 (3)
Ge1—O9^v^	1.82 (3)		
			
O1—Ho—O4	57.2 (1)	O1—Ho—O8^ii^	87.3 (2)
O1—Ho—O5^iii^	125 (2)	O1—Ho—O10^iv^	117.1 (2)
O1—Ho—O6^ii^	149 (2)	Ge1^x^—O4—Ge2	127.7 (16)
O1—Ho—O7^ii^	73.6 (2)	Ge3^iv^—O3—Ge4^viii^	165 (2)

Symmetry codes:  (i) *x*, −*y*+1/2, *z*; (ii) −*x*+1, *y*+1/2, −*z*+1; (iii) *x*, −*y*+1/2, *z*+1; (iv) *x*, *y*, *z*+1; (v) −*x*+1, *y*+1/2, −*z*; (vi) *x*, −*y*+3/2, *z*; (vii) *x*, *y*, *z*−1; (viii) *x*+1, *y*, *z*; (ix) *x*−1, *y*, *z*; (x) −*x*+1, *y*−1/2, −*z*+1.
